# Altered Endoribonuclease Activity of Apurinic/Apyrimidinic Endonuclease 1 Variants Identified in the Human Population

**DOI:** 10.1371/journal.pone.0090837

**Published:** 2014-03-04

**Authors:** Wan Cheol Kim, Conan Ma, Wai-Ming Li, Manbir Chohan, David M. Wilson III, Chow H. Lee

**Affiliations:** 1 Chemistry Program, University of Northern British Columbia, Prince George, British Columbia, Canada; 2 Laboratory of Molecular Gerontology, National Institute on Aging, National Institutes of Health, Baltimore, Maryland, United States of America; University of Pittsburgh, United States of America

## Abstract

Apurinic/apyrimidinic endonuclease 1 (APE1) is the major mammalian enzyme in the DNA base excision repair pathway and cleaves the DNA phosphodiester backbone immediately 5′ to abasic sites. APE1 also has 3′-5′ DNA exonuclease and 3′ DNA phosphodiesterase activities, and regulates transcription factor DNA binding through its redox regulatory function. The human APE1 has recently been shown to endonucleolytically cleave single-stranded regions of RNA. Towards understanding the biological significance of the endoribonuclease activity of APE1, we examined eight different amino acid substitution variants of APE1 previously identified in the human population. Our study shows that six APE1 variants, D148E, Q51H, I64V, G241R, R237A, and G306A, exhibit a 76–85% reduction in endoribonuclease activity against a specific coding region of the c-*myc* RNA, yet fully retain the ability to cleave apurinic/apyrimidinic DNA. We found that two APE1 variants, L104R and E126D, exhibit a unique RNase inhibitor-resistant endoribonuclease activity, where the proteins cleave c-*myc* RNA 3′ of specific single-stranded guanosine residues. Expression of L104R and E126D APE1 variants in bacterial Origami cells leads to a 60–80% reduction in colony formation and a 1.5-fold increase in cell doubling time, whereas the other variants, which exhibit diminished endoribonuclease activity, had no effect. These data indicate that two human APE1 variants exhibit a unique endoribonuclease activity, which correlates with their ability to induce cytotoxicity or slow down growth in bacterial cells and supports the notion of their biological functionality.

## Introduction

Apurinic/apyrimidinic endonuclease 1 (APE1) is a multi-functional protein with established roles in DNA base excision repair and in activation of the DNA-binding capacity of several transcription factors [1], [2]. Its role in DNA repair primarily entails its ability to act as an endonuclease and cleave at AP sites, generating a strand break with 3′ hydroxyl and 5′ phosphodeoxyribose termini. APE1 also acts as a 3′ phosphodiesterase/exonuclease to remove 3′ blocking groups from DNA strand break ends, generated mostly by reactive oxygen species or bifunctional DNA glycosylases that excise oxidized bases in the first step of base excision repair [1], [2]

Given the significant biological role played by APE1, it is of no surprise that investigations into single nucleotide polymorphisms (SNPs) and amino acid substitution variants have been conducted. To date, several APE1 human population variants have been identified, with the Asp148Glu (D148E) substitution being the most commonly observed, at a frequency of ∼45% [3], [4]. The D148E polymorphism has been extensively studied in molecular epidemiology studies, and there are reports suggesting association between this variant with the risk of several types of cancer [5]–[7]. However, there are a nearly equal number of reports that found no association between D148E and cancer susceptibility [8]–[10]. Novel, potentially pathogenic missense mutations have been observed in amyotrophic lateral sclerosis (ALS), more commonly known as Lou Gehrig disease [11], [12], but in another study no unique mutations were found in patients with ALS [13].

Several human APE1 population variant proteins have been expressed and purified using recombinant techniques, and their AP-DNA endonuclease activity characterized [3]. Three variants, L104R, E126D, and R237A, were found to exhibit ∼40–60% reductions in specific AP-DNA incision activity, whereas the D283A was predicted to exhibit ∼10% activity as compared to the wild-type protein [3]. In contrast, the G241R variant showed slightly enhanced DNA endonuclease activity, while both D148E and G306A had no effect on AP-DNA endonuclease and binding activities [3]. Presently, it is not clear if the altered AP-DNA endonuclease activity exhibited by some of the APE1 variants has any major impact at the cellular or organismal level, or whether there is any direct influence on susceptibility to disease.

We have described the ability of APE1 to preferentially cleave at specific single-stranded regions of the c-*myc* mRNA *in vitro*, and to regulate c-*myc* mRNA levels and half-life in cells [14]. APE1 was also found to cleave AP-site-containing single-stranded RNA [15]. Supporting a role for APE1 in RNA metabolism is evidence that APE1 interacts physically with proteins involved in ribosome assembly and RNA maturation within the cytoplasm [16].

We sought herein to analyze the RNA-cleaving activity of amino acid substitution population variants of APE1, specifically Q51H, I64V, L104R, E126D, D148E, R237A, G241R, and G306A. We show that most APE1 variants, including D148E, have a significantly diminished capacity to cleave single-stranded c-*myc* RNA substrates. Moreover, both L104R and E126D variants exhibited distinct RNA-cleaving specificity, and when over-expressed, had a cytotoxic effect in a bacterial model system.

## Materials and Methods

### Plasmid construction

pCMV6-XL5-APE1 plasmid was purchased from OriGene Technologies Inc. (Rockville, MD) and was used to generate plasmids pCMV6-XL5-L104R, pCMV6-XL5-D148E, pCMV6-XL5-E126D using PCR-based site-directed mutagenesis. pET15b-APE1 was used to generate pET15b-I64V plasmid. For generation of pET15b-Q51H, we sub-cloned a cDNA into *Nde*I and *Eco*RI restriction sites of pET15b. The following primer pairs, synthesized by Integrated DNA Technologies (IDT) Inc. (Coralville, IA), were used for PCR site-directed mutagenesis to generate all the other pET15b APE1 variants: L104R, 5′-TCA GAG AAC AAA CGA CCA GCT GAA CTT-3′, 5′-AAG TTC AGC TGG TCG TTT GTT CTC TGA-3′; E126D, 5′-CCT TCG GAC AAG GAC GGG TAC AGT GGC-3′, 5′-GCC ACT GTA CCC GTC CTT GTC CGA AGG-3′; G306A, 5′-TCC AAG GCC CTC GCC AGT GAT CAC TGT-3′, 5′-ACA GTG ATC ACT GGC GAG GGC CTT GGA-3′; D148E, 5′-TAC GGC ATA GGC GAA GAG GAG CAT GAT-3′, 5′-ATC ATG CTC CTC TTC GCC TAT GCC GTA; I64V, 5′ GCC ACA CTC AAG GTC TGC TCT TGG AAT-3′, 5′-ATT CCA AGA GCA GAC CTT GAG TGT GGC-3′. Plasmid sequences were confirmed by DNA sequencing performed at Macrogen Inc. (Seoul, Korea). Plasmids used for generation of recombinant L104R, E126D, D148E, R237A, G241R, and G306A, have been previously described [3].

### Purification of recombinant wild-type APE1 and APE1 variants

The following recombinant APE1 variants, including the WT APE1 used for comparative studies, were over-expressed in bacteria and purified as described [3]. L104R, E126D, D148E, R237A, G241R, G306A. Briefly, following ion-exchange chromatography, APE1 protein fractions were pooled, concentrated by 80% (w/v) ammonium sulfate, and then fractionated on a BioRad BioSil SEC 125-5 gel filtration column (7.8 mm×300 mm) in 50 mM Na-HEPES (pH 7.5), 5% glycerol (w/v), 0.1 mM EDTA, and 0.1 mM DTT. The flow rate was 0.25 ml/min. Proteins were detected by ultraviolet absorbance at 280 nm. APE1 proteins were dialyzed overnight against 50 mM Tris-HCl (pH 7.9), 50 mM KCl, 20% glycerol, 1 mM PMSF and 0.1 mM DTT, and stored at −70°C. Both I64V and Q51H were purified according to our previously described procedures [14], and both purified proteins were dialyzed essentially as described above for the other variants.

### Preparation of radiolabeled nucleic acids

To synthesize human c-*myc* CRD RNA corresponding to nts 1705-1792, the plasmid pGEM4Z-myc 1705-1792 was linearized and *in vitro* transcribed as described [14]. For *in vitro* endoribonuclease assay, the RNA was 5′-end radiolabeled with -[^32^P]-ATP using T4 polynucleotide kinase. For electrophoretic mobility shift assay (EMSA), internal labeling of c-*myc* CRD RNA nts 1705-1886 with α-[^32^P]-UTP (PerkinElmer, Boston, MA) was performed during *in-vitro* transcription [17].

### *In vitro* assay for endoribonuclease activity

The standard 20 µl-reaction mixture used for this assay included 2 mM DTT, 2 mM magnesium acetate, 1.0 unit of RNasin, 10 mM Tris-HCl, pH 7.4, and 25 nM (unless otherwise stated) of 5′-^32^P -radiolabeled RNA (∼5×10^4^ cpm). Reactions were incubated for 25 min at 37°C unless otherwise indicated. Forty µl of loading dye (9 M urea, 0.2% xylene cyanol, 0.2% bromophenol blue) were added to the reaction samples, and then 10 µl of reaction mixtures were subjected to electrophoresis in 8% polyacrylamide, 7 M urea gel. Gels were then dried and subjected to phosphorImaging using a Cyclone PhosphorImager.

### *In vitro* assay for AP-DNA incision

The previously established protocol for APE1 AP-DNA endonuclease assay was used with minor modifications [17]. The 18-mer oligonucleotide 5′-GTCACCGTGFTACGACTC-3′ that contain the model analog of an AP site, tetrahydrofuran (F), was used. This oligonucleotide and its complementary anti-sense strand 5′-GAGTCGTAACACGGTGAC-3′ were synthesized by IDT Inc. The oligonucleotide containing the AP site was 5′-end radiolabeled with -[^32^P]-ATP using T4 polynucleotide kinase. The reaction was stopped by heating at 95°C for 2 mins followed by hybridization to 7-fold molar excess of the anti-sense strand at room temperature for 60 mins and at 4°C overnight. The AP-DNA incision assay contains 15 µl reaction mixture consisting of 80,000 cpm (0.1 pmoles) of AP-DNA, 25 pg (0.7 fmoles) of APE1, 50 mM Tris–HCl (pH 8), 50 mM KCl, 1 mM DTT, 0.1 mM EDTA, 2 mM MgCl_2_ and 100 µg/mL bovine serum albumin. The reaction was carried out at 37°C for 3 mins. Thirty µl of loading dye (9 M urea, 0.2% xylene cyanol, 0.2% bromophenol blue) were added to the reaction samples, and then subjected to electrophoresis in 8% polyacrylamide, 7 M urea gel. Gels were then dried and subjected to phosphorImaging.

### Electrophoretic mobility shift assay

EMSA-binding buffers (5 mM Tris-Cl pH 7.4, 2.5 mM EDTA pH 8.0, 2 mM DTT, 5% glycerol, 0.1 mg/ml bovine serum albumin, 0.5 mg/ml yeast tRNA, 5 units RNasin) were prepared on ice prior to each experiment. In order to facilitate RNA denaturation and renaturation, 30 nM of [^32^P] RNA sample was heated to 50°C for 5 min and cooled to room temperature before adding to the EMSA-binding buffer. EMSA-binding buffer containing radiolabeled RNA was then incubated with purified recombinant protein in a 20-µl reaction volume at 35°C for 15 min. A total of 2 µl EMSA loading dye (250 mM Tris-Cl pH 7.4, 0.2% bromophenol blue, 0.2% xylene cyanol) was added to each reaction and 10 µl of the EMSA reaction was loaded onto an 8% native polyacrylamide gel and resolved at 25 mA for 2 hrs. Gels were then dried and subjected to phosphorImaging.

### *E.coli* Origami cell growth assays

The *E.coli* Origami cell growth assay was successfully used to indirectly assess the ribonuclease activity of RNase A and angiogenin in bacterial cells [18]. This method was also used to identify the critical amino acid residues responsible for *in vivo* ribonuclease activity of RNase A and angiogenin [18]. Competent Origami B (DE3) cells (Novagen, Mississauga, ON) were prepared using the standard protocol and kept frozen in 100 µl aliquots at −80°C. pET15b plasmids incubated on ice for 25 min before heat shock were transformed into the Origami cells by heat shock at 42°C for 75–90 secs and cooled on ice for 2 mins. After addition of 0.5 ml LB broth and incubation at 37°C with shaking for 40 mins, cells were plated onto LB agar plates containing 100 µg/ml ampicillin. Bacterial colonies were typically observed 24 hours after plating and number of colonies counted. The plasmid pGEX4T3-RNase A was used as a positive control.

For growth assay in broth, the following procedures were undertaken. Individual Origami (DE3) colonies containing the respective empty pET15-b vector or pET15b-APE1 constructs were prepared. Single 100 µL aliquots of cells were transformed with 100 ng plasmid DNA as indicated above. Upon transformation, cells were plated on LB-Ampicillin plates and grown overnight. Individual colonies of each plasmid-transformed bacteria were then selected and picked using a sterile loop and transferred to 1 ml of LB-Ampicillin broth. The cultures were then grown for one hour at 37°C. The cell counts for each of the plasmid transformed cell types were then determined using a previously established standard curve for *E. coli* cell density at OD_600_. All plasmid transformed cells were then equalized to a common cell count using LB-Ampicillin broth and subsequently transferred to 96-well tissue culture plates in triplicates. The initial OD_600_ was measured for each plasmid transformed cell type and recorded as the initial cell count at time point zero. The culture plates were then placed in an Innova40 incubator shaker set at 37°C and 200 rpm to facilitate cell growth. The micro-plates were removed from the incubator and the OD_600_ measured every hour after the initial reading for up to 12–15 hours. The data obtained was plotted in a growth curve using Kaleidagraph 4.0. Subsequently, the doubling time for each transformation was also determined using KaleidaGraph 4.0 using the equation fit parameter (e^((0.693147/m1)*m0)*m2;m1 = 5;m2 = 0.05^) which describes doubling time of bacterial cells during the exponential growth phase. The data collected from five biological replicates were then pooled and One-way ANOVA statistical analysis was performed.

## Results

### Assessing APE1 variants for ability to cleave AP-DNA

**Figure 1:**
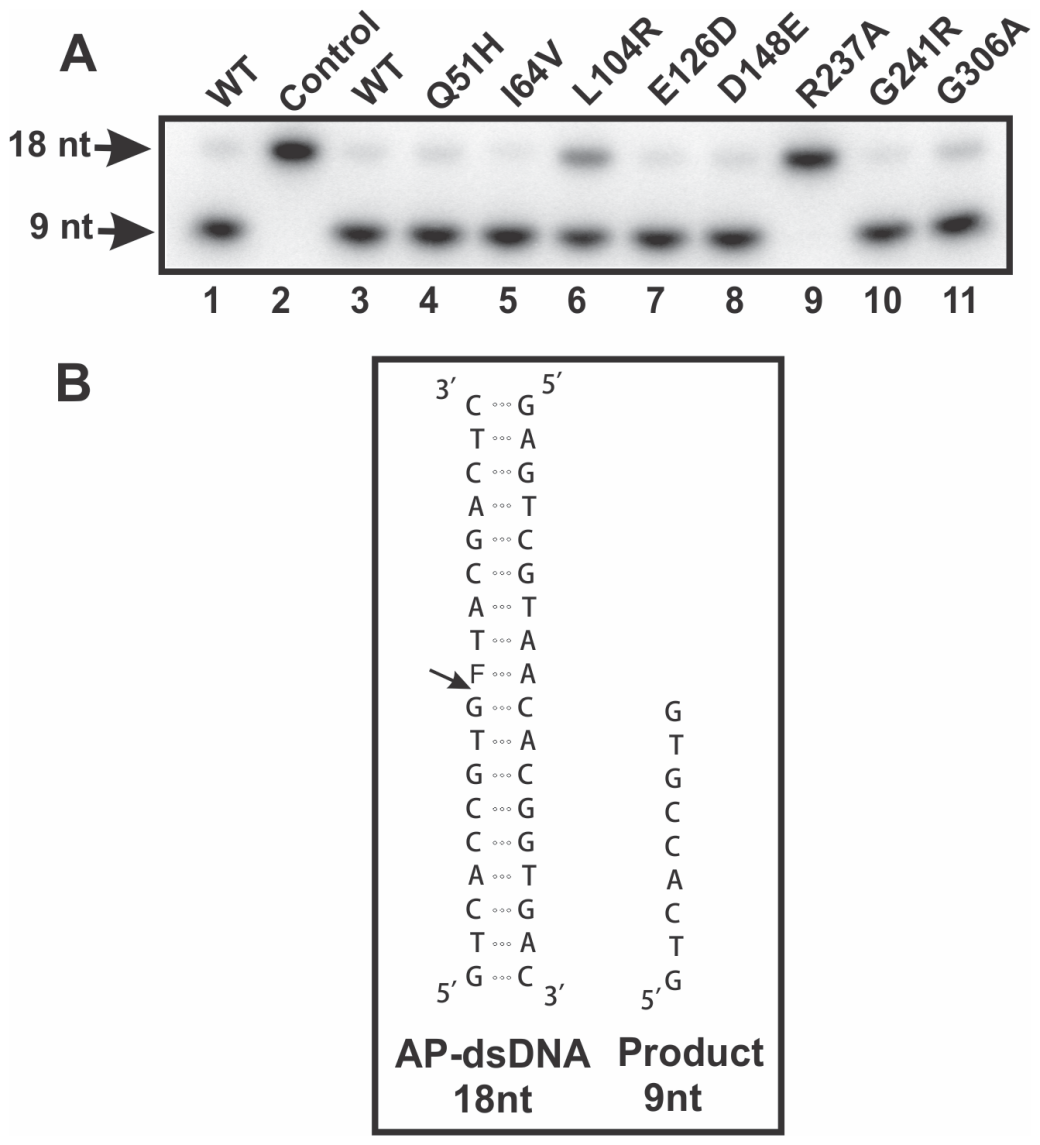
AP-DNA endonuclease activity of recombinant APE1 proteins. (**A**) AP-DNA endonuclease activities of WT APE1 (lanes 1 and 3) and APE1 variants (lanes 4–11) were assessed as described in the Experimental Procedures. Recombinant proteins (0.14 nM; lanes 1 and 3–11) were incubated with 5′-γ-^32^P-radiolabeled AP-DNA. The negative control (lane 2) has water added rather than protein samples, and the two WT APE1 (lanes 1 and 3) were from different batches of protein preparation. The 18-nt AP-DNA substrates and the 9-nt incised product are shown with arrows. (**B**) The structure and sequence of the AP-DNA substrate strand and the 9-nt single-stranded product are shown. The 18-mer oligonucleotide contains the model analog of an AP site, tetrahydrofuran (F).

As previously described, recombinant wild-type (WT) APE1 and specific APE1 variants found in the human population, specifically Q51H, I64V, L104R, E126D, D148E, R237A, G241R, and G306A, were purified to greater than 95% homogeneity [3], [19]. We first assessed the AP-DNA endonuclease activity of these proteins by measuring their ability to generate a 9-nt DNA product from a 5′^32^P-end-labeled 18-bp DNA duplex containing a model analog of an AP site, tetrahydrofuran (F) (Figure 1). In agreement with previous findings [3], [19]. Q51H, I64V, D148E, G241R, and G306A, were as equally effective as WT APE1 at cleaving the AP-DNA substrate (Figure 1A and Table 1). R237A showed complete loss of AP-DNA endonuclease activity (Figure 1A and Table 1), which is consistent with this protein displaying instability and a severely diminished incision capacity [3]. We found the L104R variant to have ∼26% decreased AP-DNA endonuclease activity (Figure 1A and Table 1), which is also consistent with previous results [3]. Notably, the E126D variant exhibited no change in endonuclease function here, whereas it was previously described to display ∼40% reduction in incision activity [3]. This discrepancy may be attributed to the differences in the buffer conditions used, as we employed Tris-HCl buffer containing 50 mM KCl, 1 mM DTT, 0.1 mM EDTA, 2 mM MgCl_2_, and 100 µg/ml BSA, while the previous study utilized HEPES-KOH buffer (pH 7.5) containing 50 mM KCl, 10 mM MgCl_2_, 10% glycerol, 0.05% Triton X-100, and 100 µg/ml BSA [3]. Alternatively, the discrepancy may be due to slight differences in the sequence of DNA substrate used [3].

**Table 1:** Summary of Endoribonuclease and Abasic dsDNA Incision Activities of APE1 Population Variants.

APE1 populationvariants	Abasic dsDNA incision^a^(% of WT)	Abasic dsDNA incision activity^b^(% of WT) ± S.D.	Endoribonuclease activity against c-*myc* CRD^c^(% of WT) ± S.D.
Q51H	ND	101±2.10	23.1±2.5
I64V	ND	103±4.40	15.6±3.0
L104R	56	74±1.50	NA^d^
E126D	60	102±3.10	NA^d^
D148E	94	101±5.60	23.3±4.4
R237A	35	0.1±0.01	6.0±2.0
G241R	108	101±4.05	24.3±2.4
G306A	107	97±2.20	20.4±3.2

**Figure 2:**
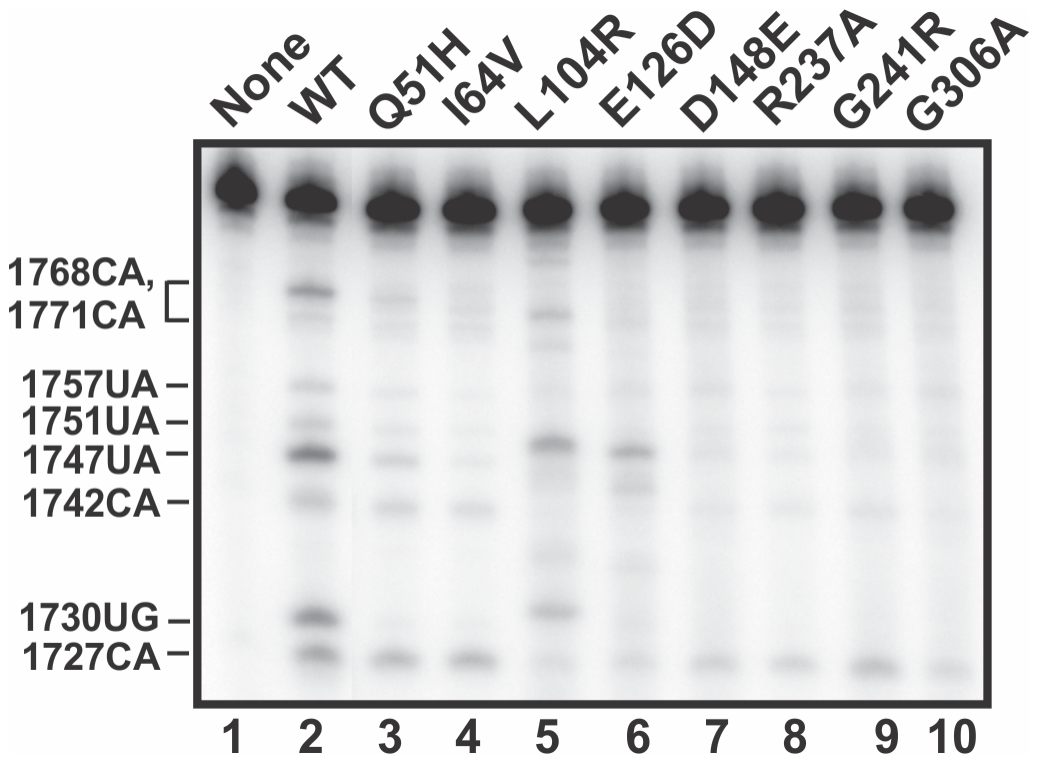
APE1 variants display reduced or altered endoribonuclease activity. Endoribonuclease assay was carried out on c-*myc* CRD RNA with WT APE1 and the indicated variants as described in the Experimental Procedures. Recombinant proteins (1.4 µM; lanes 2 to 10) were incubated with 25 nM of 5′-γ-^32^P-radiolabeled c-*myc* nts 1705-1792 CRD RNA in a total reaction volume of 20 µl for 25 mins at 37^o^C. Numbers on the left indicate the cleavage sites generated by the WT APE1 protein.

**Figure 3:**
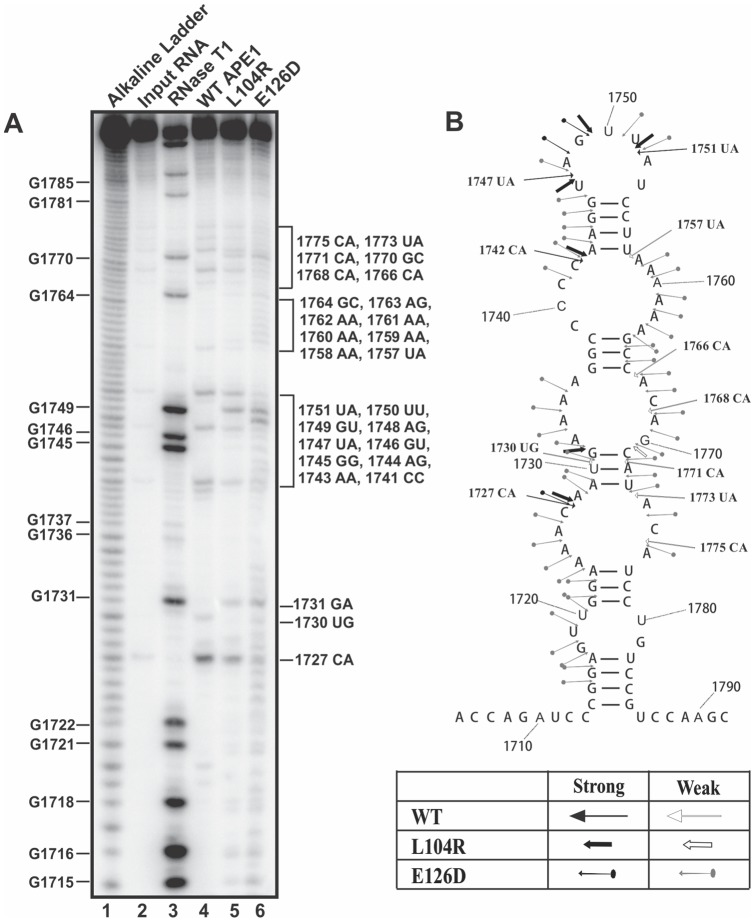
Mapping of the altered RNA cleavage sites generated by L104R and E126D APE1 variants. (**A**) Recombinant WT APE1 (lane 4), variant L104R (lane 5), and variant E126D (lane 6), each at 1.4 µM, were incubated with 350 fmoles of 5′-^32^P-radiolabeled c-*myc* CRD RNA for 25 min at 37°C in a total volume of 20 µl. Samples were run on a 12% polyacrylamide/7 M urea gel. For reference, an alkaline hydrolysis ladder was generated (lane 1) and RNase T1 digest of the c-*myc* CRD RNA (lane 3) was performed. Numbering on the left indicates guanosine residue sites cleaved by RNase T1 under denaturing conditions. Numbering on the right indicates sites cleaved by WT APE1 and the APE1 variants L104R and E126D. (**B**) Secondary structure of c-*myc* CRD RNA and the cleavage sites generated by WT APE1 (underlined) and APE1 variants L104R and E126D. The box at the bottom of the figure indicates the strong and weak cleavage sites generated by the endoribonucleases.

ND denotes not determined; NA denotes not applicable.

a Information obtained from the literature [3].

b Data obtained from Figure 1A and two other independent experiments.

c Data obtained from Figure 2 and two other independent experiments.

d Altered cleavage sites was observed. See Figures 2 and 3.

### Assessing APE1 variants for ability to cleave c-*myc* CRD RNA

We next compared the ability of the purified recombinant APE1 variants to cleave the 88-nt ^32^P-c-*myc* coding region determinant (CRD) RNA substrate relative to WT APE1 [14]. Using the shorter version of the c-*myc* CRD RNA substrate, which was either 5′-radiolabeled with ^32^P-γ-ATP or uniformly labeled with ^32^P-α-UTP, we have specifically demonstrated the endoribonuclease activity of the recombinant WT APE1 protein [11]. In this study and in our previous studies, 0.1–1.4 µM of APE1 was used to assess endoribonuclease activity [14], [17], [20]. The requirement for micromolar range concentrations to demonstrate endoribonuclease activity *in vitro* is not unusual, as this has been shown for other endoribonucleases including PMR-1, IRE1, ARD-1, angiogenin, SMG6, and Rrp44 [20]. Furthermore, a correlation with the ability to influence the steady-state level of the target mRNA in cells for some of these endoribonucleases has been documented [20]. As shown in Figure 2 and consistent with previous findings [14], WT APE1 specifically cleaved c-*myc* CRD RNA at the following sites: 1727CA, 1730UG, 1742CA, 1747UA, 1751UA, 1757UA, 1768CA, 1771CA. However, most variants, particularly D148E, R237A, G241R, G306A, I64V, and Q51H, exhibited a significant reduction (76–85%) in their ability to cleave c-*myc* CRD RNA as compared to the WT APE1 protein (Figure 2 and Table 1). This conclusion is based on the intensity of the major bands at cleavage sites 1730UG, 1742CA, 1747UA, 1751UA, 1757UA, 1768CA and 1771CA. Interestingly, the L104R and E126D variants appeared to cleave c-*myc* CRD RNA in a unique pattern compared to the WT APE1 (Figure 2).

We next mapped the distinct cleavage sites generated by both the L104R and E126D variant. As shown in Figure 3, we found L104R to specifically cleave c-*myc* CRD RNA at the following sites: 1727CA, 1731GA, 1742CA, 1747UA, 1749GU, 1751UA, 1768CA, 1770GC, 1771CA, 1773UA, and 1775CA. It is important to point out that L104R was capable of cleaving after guanine bases, as illustrated by cleavage at 1731GA, 1749GU, and 1770GC. For E126D, the prominent cleavage sites were at 1727CA, 1731GA, 1748AG, and 1749GU (Figure 3). In addition, laddering patterns were observed at regions 1715-1734, 1741-1751, and 1758-1775, suggestive of exoribonucleolytic decay by E126D.

We have previously demonstrated that WT APE1 also preferentially cleaves at UA, UG, and CA sites on additional RNA substrates, including CD44, orf1b, orf3, spike, miR-21, and miR-10b [21]. Hence, we expect that both L104R and E126D would be able to cleave other RNA substrates *in vitro*, but with reduced efficiency. To further characterize the RNA cleavage activity of these variants, we compared WT APE1 with L104R and E126D for their ability to cleave an abasic RNA substrate (34F AP-RNA; Figure S1). Consistent with the finding using the c-*myc* RNA substrate, we found that the cleavage sites generated by WT APE1, i.e., 4CA, 8UG, and 18UA, were reduced in the L104R- and E126D-treated samples (lanes 5–10). Surprisingly, both L104R and E126D variants had significantly enhanced ability to cleave at an abasic site in RNA compared to the WT protein.

### Assessing APE1 variants for ability to bind c-*myc* CRD RNA

**Figure 4:**
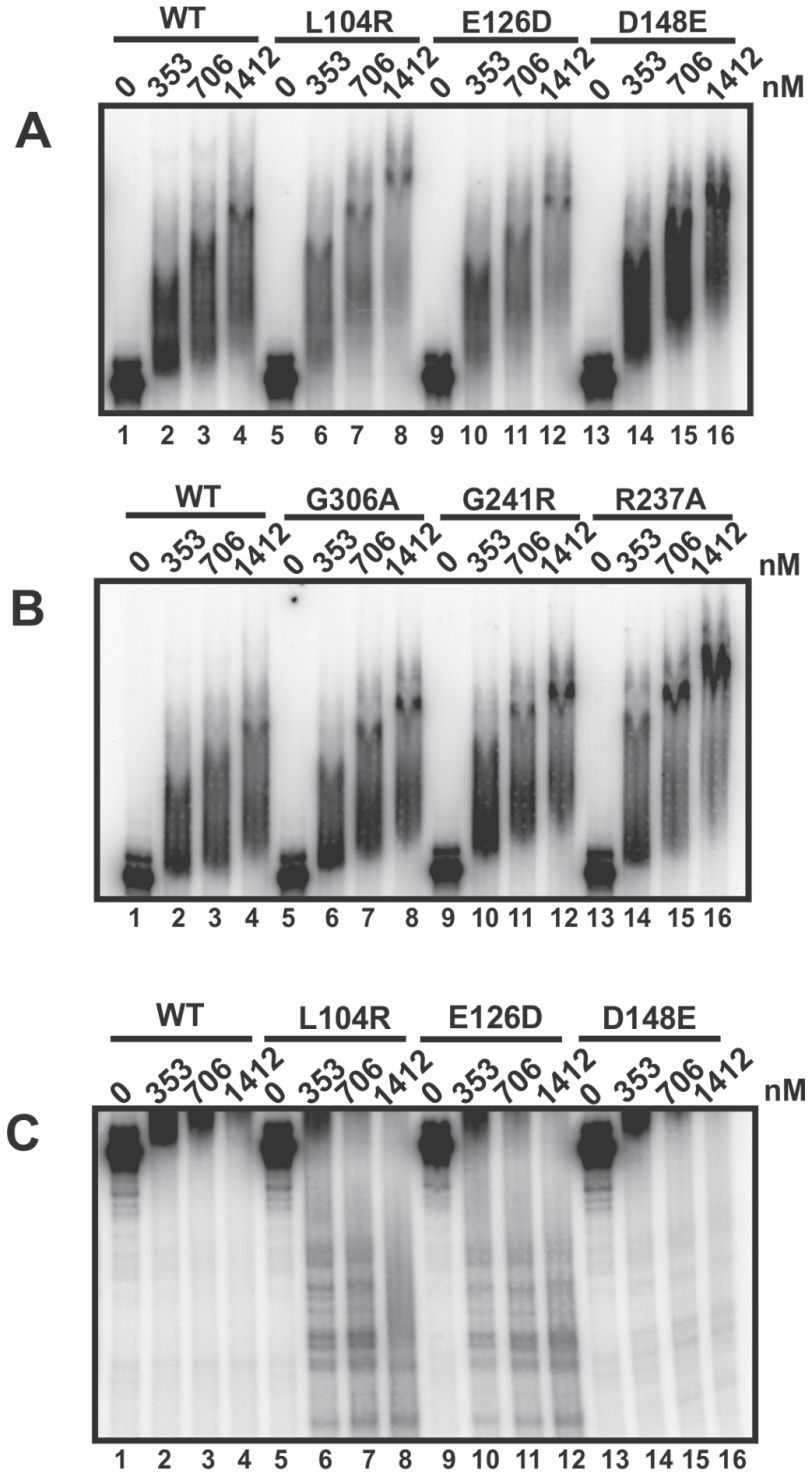
Ability of APE1 and its population variants to bind c-*myc* CRD RNA. Increasing amounts of APE1 recombinant proteins were incubated with 50 nM of internally-labeled α-[^32^P]-UTP c-*myc* CRD RNA (nts 1705-1886) in a total binding volume of 20 µl for 15 min at 35^o^C as described in the Experimental Procedures. (**A**) EMSA showing the binding of WT APE1 (lanes 2–4), APE1 variants L104R (lanes 6–8), E126D (lanes 10–12), and D148E (14–16) to c-*myc* RNA at a final concentration ranging from 353 to 1412 nM. (**B**) A standard EMSA with WT APE1 (lanes 2-4) and APE1 variants G306A (lanes 6 to 8), G241R (lanes 10 to 12), and R237A (lanes 14 to 16). (**C**) EMSA showing the bottom half of the gel from (**A**) above.

The reduced endoribonuclease activity of the APE1 variants could be due to a reduced ability to bind RNA and/or a deficiency in catalysis. To assess the capacity of the APE1 variants to bind c-*myc* CRD RNA, we performed electrophoretic mobility shift assays (EMSA). We have previously optimized the conditions for binding of WT APE1 to c-*myc* CRD RNA and found that at a concentration range from 0 to 1412 nM, WT APE1 exhibits essentially linear binding [17]. We reasoned that if there were differences in the binding affinity amongst the recombinant APE1 variants, these would most likely be observed in this concentration range. The results in Figure 4A and 4B show that L104R, E126D, D148E, G306A, G241R, and R237A all display RNA binding affinities comparable to WT APE1. However, we noticed a two-fold decrease in intensity of RNA-protein complexes in the L104R and E126D lanes, suggestive of decreased RNA-protein complex formation or stability (lanes 6-8 and lanes 10–12, Figure 4A). When we examined the lower portion of the gel in Figure 4A (i.e., below the unbound substrate), we noticed distinct bands generated only by L104R and E126D, and not by the WT protein or the D148E variant (Figure 4C). These smaller RNA fragments suggest that both L104R and E126D variants cleave and degrade c-*myc* CRD RNA in the EMSA buffer, despite the presence of the RNase inhibitor, RNasin.

**Figure 5:**
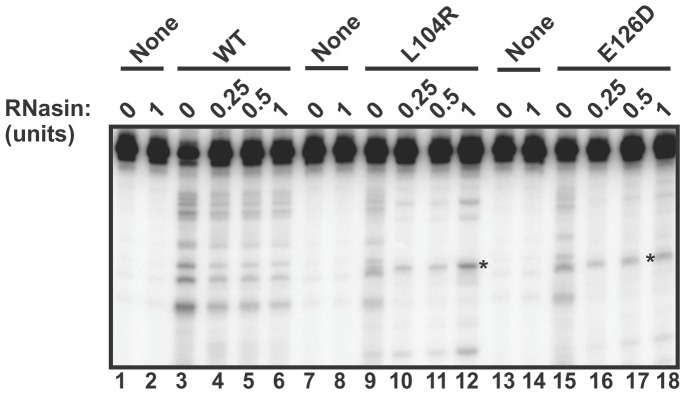
The distinct endoribonuclease activity of L104R and E126D APE1 variants is resistant to RNasin, an RNase inhibitor. WT APE1, L104R or E126D (1.4 µM) were tested against 25 nM of 5′-γ-^32^P-radiolabeled c-*myc* nts 1705-1792 CRD RNA in a total reaction volume of 20 µl for 25 mins at 37°C. RNasin, at 0.25 to 1 U, was added to the specified samples as denoted. Asterisks indicate cleavage sites generated by L104R and E126D that are resistant to RNasin inhibition.

We next assessed the sensitivity of both L104R and E126D variants to RNasin in a c-*myc* CRD RNA endonucleolytic assay as performed above in Figure 2. All cleavage bands generated by WT APE1 on c-*myc* CRD RNA were gradually reduced in the presence of increasing RNasin (0.25–1.0 unit) (lanes 3–6, Figure 5). In contrast, some distinct bands generated by either L104R or E126D remained even up to 1 unit of RNasin (lanes 9–12 and lanes 15–18, Figure 5), indicating that the unique RNA-cleaving ability of both L104R and E126D is resistant to RNasin. The slightly more intense band in the L104R lane containing 1 unit of RNasin (lane 12) could be the result of slightly higher loading of the RNA substrate.

### Over-expression of L104R and E126D are toxic to *E.coli* Origami cells

A genetic bacteria-based system that uses *E.coli* Origami cells has been successfully employed to identify residues that are essential for the function of endoribonucleases, such as RNase A and angiogenin [18]. This system takes advantage of the ability of Origami cells to allow proper folding of ribonucleases into functional enzymes. When expressed, these ribonucleases have been shown to cause cell death in Origami cells, presumably due to their enzymatic activity to cleave bacterial RNA [18]. We used this system to assess whether the WT APE1 protein and the variants are functional as ribonucleases in cells.

**Figure 6:**
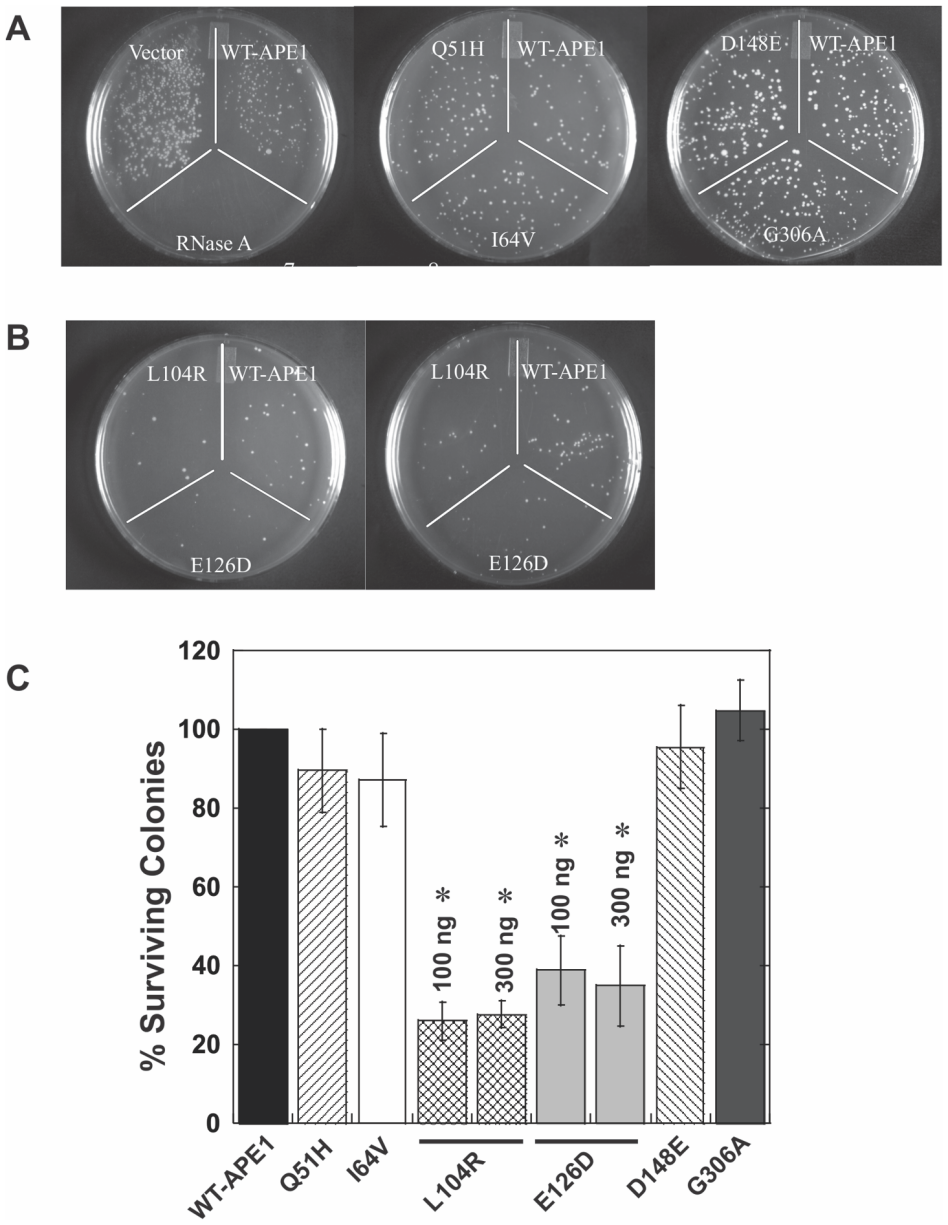
Effect of expressing APE1 variant proteins on colony formation in *E.coli* Origami cells. (**A**) Competent *E.coli* Origami (DE3) cells were transformed with 300 ng of plasmid vector pET15b, 300 ng pET15b-WT APE1, or 100 ng pGEX4T3-RNase A (left plate). Cells were plated onto a LB-ampicillin agar plate and incubated for 18 to 24 hrs at 37°C. In separate experiments, 300 ng pET15-Q51H, pET15b-WT APE1, pET15b-I64V, pET15b-D148E, or pET15b-G306A plasmid was transformed into Origami cells (middle and right plates). (**B**) Competent Origami cells were transformed either with 100 ng (left plate) or 300 ng (right plate) pET15b-WT APE1, pET15b-L104R, or pET15b-E126D. (**C**) Number of colonies formed after transformation of Origami cells with 100 ng or 300 ng plasmid DNA as described in (**A**) and (**B**) were counted, and the data were pooled with another two sets of separate experiments. Results show the percent surviving colonies after transformation for each plasmid. For comparison, the number of colonies formed upon transformation with the pET15b-WT APE1 was taken as 100%. Error bars are standard deviation and One-way ANOVA analysis show results to be statistically significant (*, p = 0.001).

We initially included IPTG in our experimental procedure, but found this compound to be extremely toxic to bacterial growth for cells transformed with the APE1 expression plasmids (unpublished observation). Hence, in the experiments described below, protein production was not induced and was due to leaky expression. RNase A was used as a positive control, and Figure 6A indicates that no cells grew when transformed with pGEX4T3-RNase A due to its strong ribonuclease activity. When cells were transformed with pET15b-WT APE1, there was about 50% reduction in the number of colonies compared to the pET15b vector-transformed cells (left plate, Figure 6A). Thus, our results confirmed that the *E.coli* Origami cells can be used to indirectly assess the ribonuclease activity of RNase A in cells, and suggest that we can use this method to indirectly study the endoribonuclease activity of APE1 in cells.

We next compared the colony formation ability of Origami cells transformed with pET15b plasmids harboring a cDNA for each of the APE1 variants. We found that 300 ng of pET15b-Q51H or pET15b-I64V (the middle plate in Figure 6A) and 300 ng of pET15b-D148E or pET15b-G306A (the right plate in Figure 6A) had a similar effect on colony formation as pET15b-WT APE1. We also tested 100 ng of the above plasmids carrying the APE1 variants and found no differences in their effect on Origami colony formation as compared to the WT APE1 (unpublished observation). Conversely, we found that the number of colonies formed was significantly reduced when transformation was done using 100 ng (left plate, Figure 6B) or 300 ng (right plate, Figure 6B) of either pET15b-E126D or pET15b-L104R relative to WT APE1. Figure 6C shows the pooled quantitative data, which were derived from counting the number of colonies on plates for three separate experiments conducted with the different plasmid vectors at the indicated amount of DNA.

**Figure 7:**
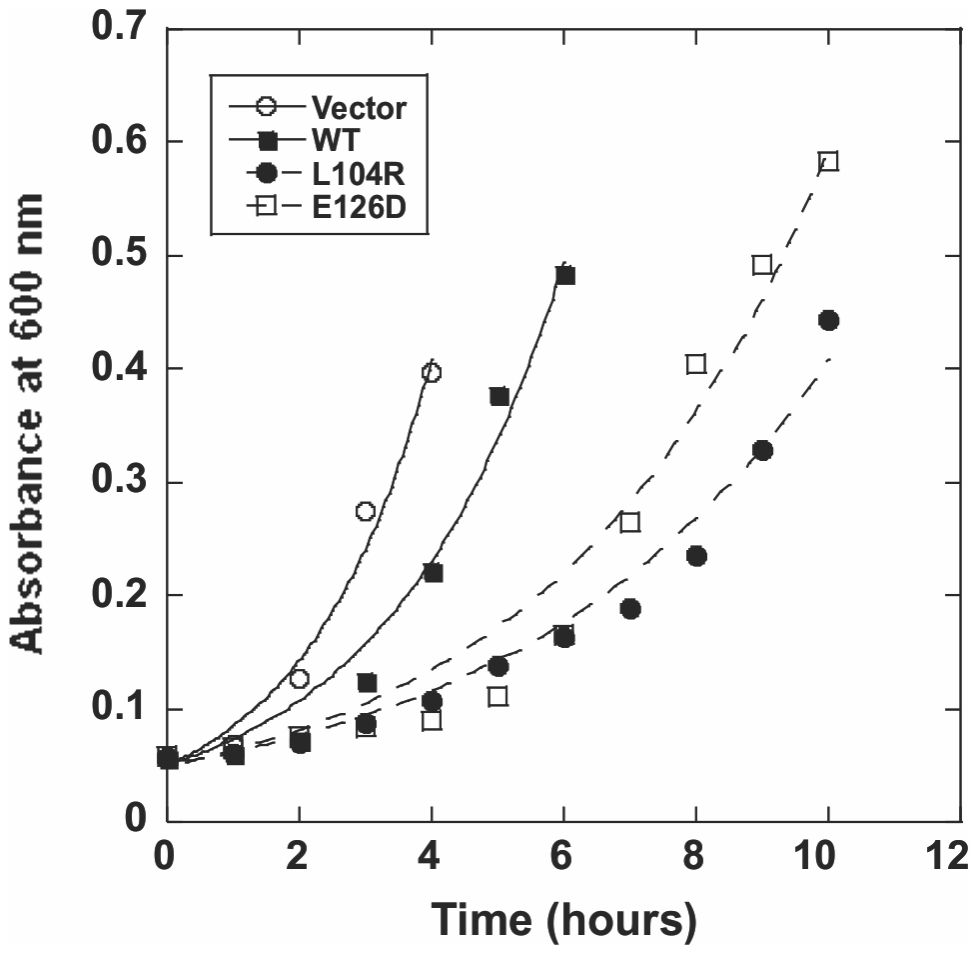
Effect of over-expressing L104R and E126D APE1 variants on the growth of *E.coli* Origami cells in culture. Individual colonies of *E.coli* Origami cells obtained upon transformation of cells with pET15b vector, pET15b-WT APE1, pET15b-L104R, or pET15b-E126D, were picked and grown overnight at 37°C in LB-Ampicillin broth. Using an established standard curve for *E. coli* cell density at OD_600_, all grown bacteria were equalized to a common cell count in LB-Ampicillin broth and subsequently transferred to 96-well tissue culture plates in triplicates. The initial OD_600_ was measured and taken as time point zero. The culture plates were then placed in a shaker incubator at 37°C, and the OD_600_ was taken every hour after the initial reading for a total of 12–15 hours. The data shown is a representative growth plot from five independent experiments.

To further confirm the above results, which were based on colony formation, we assessed the growth of Origami cells in liquid culture following transformation with the different plasmids. Picked colonies were grown up in LB for up to 12–15 hours, with absorbance readings taken every hour. The growth of Origami cells during exponential growth phase was plotted as shown in Figure 7. It was clear that cells transformed with plasmid DNA expressing WT APE1 displayed a longer doubling time than cells transformed with the pET15b vector (Figure 7). Moreover, both the L104R- and the E126D-transformed cells exhibited even slower growth when compared to the pET15b-WT APE1-transformed cells. The doubling time of these bacteria was determined from five biological replicates and is summarized in Table 2. Our results show that pET15b-Q51H (1.52±0.09), pET15b-I64V (1.58±0.08), pET15b-D148E (1.63±0.10), and pET15b-G306A (1.81±0.08) had an effect on the doubling time of Origami cells in culture that was similar to pET15b-WT APE1 (1.78±0.17) (p>0.05, Tukey's Test, One-way ANOVA). Conversely, pET15-L104R (2.84±0.15) and pET15b-E126D (2.48±0.19) significantly increased the doubling time in comparison to pET15b-WT APE1 (p<0.001, Tukey's Test, One-way ANOVA). The cell broth assay therefore agrees with the results from the Origami colony formation assay, indicating that both L104R and E126D are more potent than WT APE1 at reducing cell growth via a mechanism that likely involves RNasin-resistant endoribonuclease activity.

**Table 2:** Effect on Growth of Origami Cells Upon Transformation with Human APE1 Population Variants.

pET15b plasmids	Doubling time (hours)^a^
Vector	1.22±0.16
WT APE1	1.78±0.17
Q51H	1.52±0.09
I64V	1.58±0.08
L104R	2.84±0.15^b^
E126D	2.48±0.19^b^
D148E	1.63±0.10
G306A	1.81±0.08

a The doubling time obtained from Figure 7 using KaleidaGraph 4.0.

b P<0.001 when compared with WT APE1, Tukey's test, One-way ANOVA.

## Discussion

We previously demonstrated that APE1 preferentially cleaves at UA, CA, and UG dinucleotides in single-stranded regions of RNA [14], [17], [21]. APE1 was also shown to cleave AP-site-containing single-stranded RNA [15], [17]. Towards understanding the biological significance of the RNA-cleaving function of APE1, we generated and purified a panel of APE1 variants that are found in the human population and assessed them for their ability to cleave ^32^P-labeled c-*myc* CRD RNA, a substrate that we previously used to identify the endoribonuclease activity of native APE1 [14]. We found that most APE1 variants, including the most common variant D148E, showed significantly diminished ability to cleave c-*myc* CRD RNA. Surprisingly, we found that both L104R and E126D variants were able to cleave c-*myc* RNA at sequences that are distinct from WT APE1, and this altered activity correlates with the ability of these proteins to cause cytotoxicity or slow down growth in bacterial cells.

We observed that in addition to the regular cleavage sites, such as 1727CA, 1742CA, 1747UA, 1751UA, 1768CA, 1771CA, 1773UA and 1775CA, the L104R variant prominently cleaved 3′ of single-stranded guanosine residues at 1731GA, 1749GU, and 1770GC (Figure 3). Using molecular modeling, it was previously shown that L104 is positioned in the loop between β–sheet number 2 and α–helix number 3, which is adjacent to the general nucleic acid binding/recognition site [3]. Upon substitution with an arginine, the normal hydrophobic interactions between L104 and its neighbors (L72, L108, and W119) are predicted to be diminished, possibly resulting in further attraction of the RNA substrate into the newly formed positively charged local region. We found that E126D substitution in APE1 resulted in unusual cleavage patterns on c-*myc* CRD RNA as well. In addition to the prominent cleavage sites at 1731GA, 1748AG, and 1749GU, laddering patterns were observed at regions of 1715-1734, 1741-1751, and 1758-1775, which was suggestive of exoribonucleolytic decay (Figure 3). According to molecular modeling, E126 is positioned in a loop between β–sheet number 3 and 4 [3]. Although it appears that E126D substitution is a relatively minor modification in that the negative charge is preserved and the side chain is only shortened by one –CH_2_-group, the crystal structure of APE1-AP-DNA complex did show that E126 is in close proximity to the DNA phosphate backbone [12]. Perhaps, with the shortening of one –CH_2_-group, the phosphate backbone of the RNA substrate can become more amendable leading to the observed exoribonucleolytic activity. The addition of one –CH_2_-group as seen in D148E substitution led to diminished RNA-cleaving activity of APE1 (Figure 3).

The diminished endoribonuclease activity of D148E, Q51H, I64V, G241R, R237A, and G306A, is due to a defect in enzymatic catalysis and not in substrate binding, because all the APE1 variants were found to bind c-*myc* RNA equally well compared to WT APE1 (Figure 4). We currently do not know the precise mechanistic basis for the altered endoribonuclease activity of the APE1 variants. Future structural studies of WT and variant APE1 proteins, ideally in complex with an RNA substrate, would provide insights into how APE1 binds and cleaves RNA, as well as provide useful information about protein engineering of ribonucleases.

Another interesting finding from this study was that the RNA-cleaving activities of L104R and E126D are resistant to the common RNase inhibitor, RNasin. This prompted us to investigate the biological effect of over-expressing both variants in cells. We adopted the bacterial Origami cells method previously used to indirectly assess ribonuclease activities of WT and mutant forms of both RNase A and angiogenin [18]. Using the previously described plate method [18] and a culture method, we found that cells transformed with either L104R or E126D had diminished cell growth when compared with cells transformed with WT APE1 (Figures 6 and 7). Surprisingly, we found that the APE1 variants D148E, G306A, Q51H, and I64V, which showed diminished endoribonuclease activity (Figure 2 and Table 1), had comparable growth effects to those observed with WT APE1 (Figure 6). A similar lack of effect was also seen for angiogenin mutants with decreased ribonucleolytic activity [18]. We postulate that this outcome is due to the insensitivity of the cell method to detect loss-of-function mutations as opposed to gain-of-function mutations as in the case of L104R and E126D variants. Our data validate the Origami cell system for assessing ribonuclease activity [18] and suggest that the ribonuclease activity of WT APE1 and its two variants L104R and E126D can be indirectly demonstrated in bacterial cells.

It should be mentioned that some of the human APE1 variants studied here were based on an earlier report that described APE1 missense mutations in a small group of patients with ALS in the United States [22]. Two subsequent studies with larger groups of patients in the United Kingdom found no such missense mutations, except for the two polymorphic D148E and Q51H substitutions [6], [11], raising doubt about the validity of the initial observations and the association of APE1 with this disease. Our findings with D148E are nevertheless intriguing. In particular, while retaining its full AP-DNA endonuclease and binding activities [3], [19], the variant has a significantly diminished endoribonuclease activity. An obvious question to address going forward is whether this dramatic loss in biochemical activity of D148E has any relevance in disease risk [5]–[7]. Equally important is whether L104R or E126D has any effect on mammalian cell growth in culture, at the organismal level, and altered disease susceptibility.

As a multifunctional protein, it has been rather challenging to determine which biochemical activity of APE1 is responsible for the phenotypic changes observed upon modulation of its expression. The combined knowledge from the present and previous studies on the distinct biochemical activities of APE1 mutants, such as the D148E and D283N [17], should assist us in properly investigating the contributions of each biochemical function of APE1 in cells. This study also shows that the endoribonuclease activity of APE1 and its population variants correlates with their cytotoxic effect on bacterial cells, suggesting that RNA cleaving activity of APE1 and its variants can be functional in cells. Future studies are required to directly determine whether the endoribonuclease activity of APE1 and its variants are functional in mammalian cells and what phenotype they produce, if any, as a consequence of this unique biochemical activity.

## Supporting Information

Figure S1 **Mapping of the RNA cleavage sites on 34F AP-RNA substrate generated by WT APE1, L104R and E126D APE1 variants.** (**A**) Recombinant WT APE1 (lane 4) at 1.4 µM, and variant L104R (lanes 5–7), and variant E126D (lanes 8–10), each at 1.4, 3, and 6 µM, were incubated with 350 fmoles of 5′-^32^P-radiolabeled 34F AP-RNA for 30 minutes at 37°C in a total volume of 20 µl. Samples were run on a 12% polyacrylamide/7 M urea gel. For reference, an alkaline hydrolysis ladder was generated (lane 1) and RNase T1 (lane 12) and RNase A (lane 11) digest of the 34F AP-RNA were performed. Numbering on the left indicates guanosine residue sites cleaved by RNase T1 under denaturing conditions, as well as sites cleaved by RNase A. (**B**) Secondary structure of 34F AP-RNA and the cleavage sites generated by WT APE1 and APE1 variants L104R (left panel) and E126D (right panel). The box at the bottom of the figure indicates the strong and weak cleavage sites generated by APE1 and its variants. The oligonucleotide contains the model analog of an AP site, tetrahydrofuran (F). The asterisk in both (**A**) and (**B**) indicates the abasic cleavage site.
(TIF)
