# Supplementary figures and images for: Altered Endoribonuclease Activity of Apurinic/Apyrimidinic Endonuclease 1 Variants Identified in the Human Population
Source: PLoS One. 2014 Mar 4;9(3):e90837. doi: 10.1371/journal.pone.0090837 (PMC3942487; doi:10.1371/journal.pone.0090837)

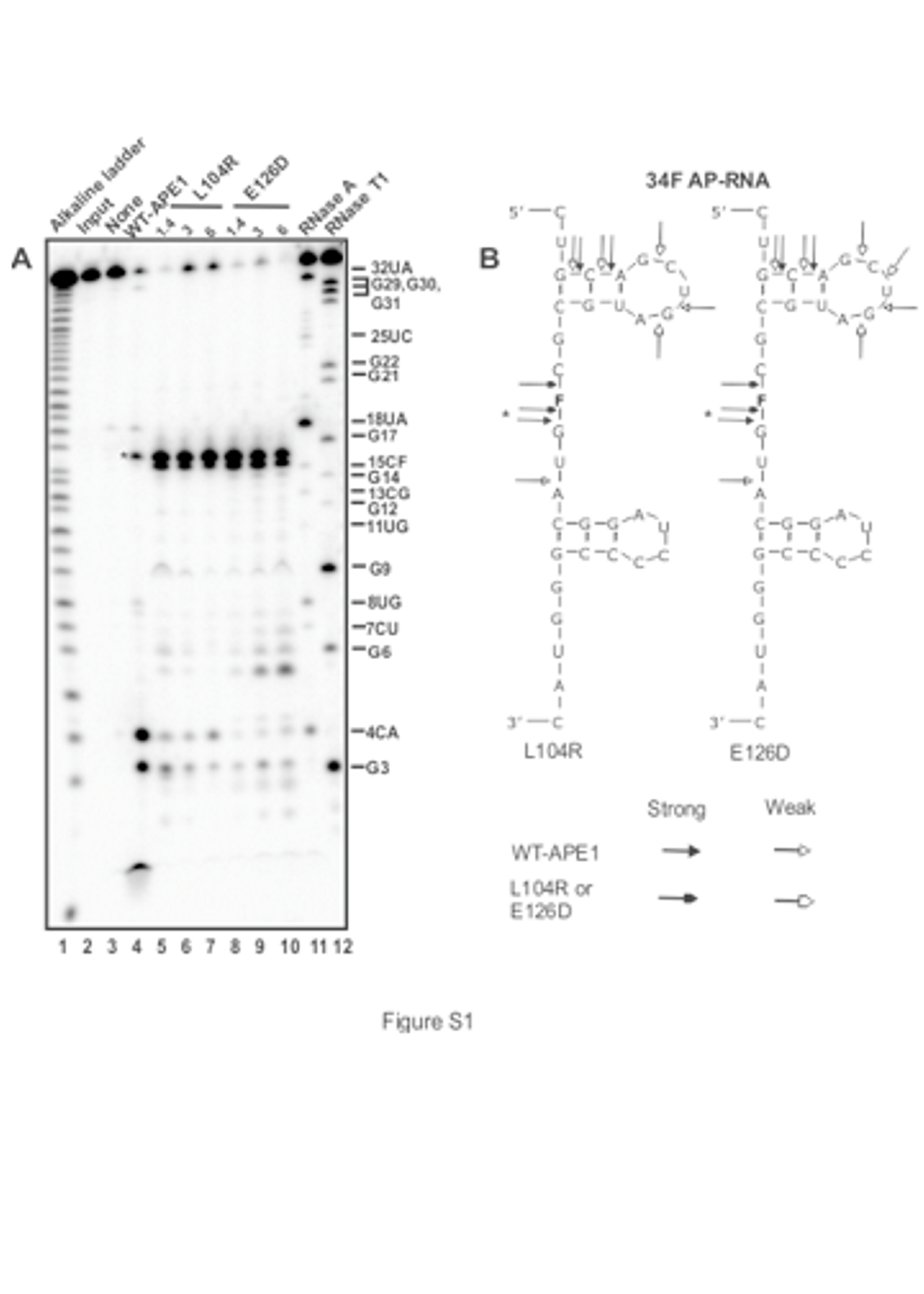

Supplement: Figure S1 — Mapping of the RNA cleavage sites on 34F AP-RNA substrate generated by WT APE1, L104R and E126D APE1 variants. (A) Recombinant WT APE1 (lane 4) at 1.4 µM, and variant L104R (lanes 5–7), and variant E126D (lanes 8–10), each at 1.4, 3, and 6 µM, were incubated with 350 fmoles of 5′-32P-radiolabeled 34F AP-RNA for 30 minutes at 37°C in a total volume of 20 µl. Samples were run on a 12% polyacrylamide/7 M urea gel. For reference, an alkaline hydrolysis ladder was generated (lane 1) and RNase T1 (lane 12) and RNase A (lane 11) digest of the 34F AP-RNA were performed. Numbering on the left indicates guanosine residue sites cleaved by RNase T1 under denaturing conditions, as well as sites cleaved by RNase A. (B) Secondary structure of 34F AP-RNA and the cleavage sites generated by WT APE1 and APE1 variants L104R (left panel) and E126D (right panel). The box at the bottom of the figure indicates the strong and weak cleavage sites generated by APE1 and its variants. The oligonucleotide contains the model analog of an AP site, tetrahydrofuran (F). The asterisk in both (A) and (B) indicates the abasic cleavage site. (TIF) [file pone.0090837.s001.tif]
